# An Alternative Approach for the Rehabilitation of a Complete Denture Patient With Parkinson’s Disease

**DOI:** 10.7759/cureus.66406

**Published:** 2024-08-07

**Authors:** Madhu Priya, Surekha A Dubey, Jahnavi P Gorripati

**Affiliations:** 1 Department of Prosthodontics, Sharad Pawar Dental College and Hospital, Datta Meghe Institute of Higher Education and Research, Wardha, IND

**Keywords:** debilitating disorders, neurological disorder, neutral zone technique, complete denture prosthesis, parkinson' s disease

## Abstract

Parkinson's disease (PD) affects one to two out of every 1,000 individuals. PD, being age-related, is affecting a percentage of those over and around the sixth decade. Lewy bodies containing α-synuclein and a reduction in dopaminergic neurons in the substantia nigra, which impairs the region's capacity to promote voluntary movements, are the main neuropathological findings. The three main symptoms of PD are stiffness, bradykinesia, and tremor. Treatment of edentulous conditions in patients with PD becomes a challenge due to decreased neuromuscular coordination and decreased mobility. This case report outlines a 64-year-old male patient with complete edentulism suffering from PD. Complete denture fabrication was done using the concepts of the neutral zone and denture characterization. Significantly, it not only improves the stability of the dentures but also establishes good denture aesthetics.

## Introduction

Age is the single biggest reason for the start and progression of Parkinson’s disease (PD). Numerous cellular mechanisms caused by aging and changes in cellular function are factors that affect neurodegeneration and also increase the risk of PD [1]. It may worsen with age if compensatory systems fail to function and age-related somatic damage accumulates [2]. Reflexivity, bradykinesia, tremors, and issues with gait and balance are frequent clinical examination findings in older individuals without a clear neurological condition that are indicative of mild Parkinsonian symptoms [3].

The patient's decreased adaptive abilities, increased saliva, increased tremors, and poor muscular control, make prosthodontic treatments challenging to execute and jeopardize denture retention [4]. Patients with PD may have orofacial characteristics such as a mask-like face from reduced muscular tone, poor speech and articulation, a dry mouth, and difficulties swallowing with saliva dribbling from the corners of the mouth. Depression, cognitive issues, and a loss of muscle control are the causes of poor dental hygiene. Inadequate oral hygiene raises the risk of dental caries, periodontal disease, and premature tooth loss. For these patients, oral rehabilitation can be difficult for the dentist since the patient's ability to use their mouth musculature to regulate the denture is crucial to the procedure's effectiveness [5]. Thus, in the edentulism state, complete denture rehabilitation should be done with the aim of improving denture retention and stability without compromising the aesthetics, function, or phonetics of the patient. Various rehabilitation methods have been described in the literature for complete dentures in patients with PD, such as utilizing the neutral zone concept and monoplane teeth, reducing clinical visits [6].

This case report outlines the complete denture rehabilitation of an edentulous patient suffering from age-related PD with decreased neuromuscular coordination, rigidity of facial muscles, and decreased salivation. The neutral zone concept and high-impact strength complete dentures have been employed to facilitate stability, strength, and aesthetics.

## Case presentation

**Figure 1:**
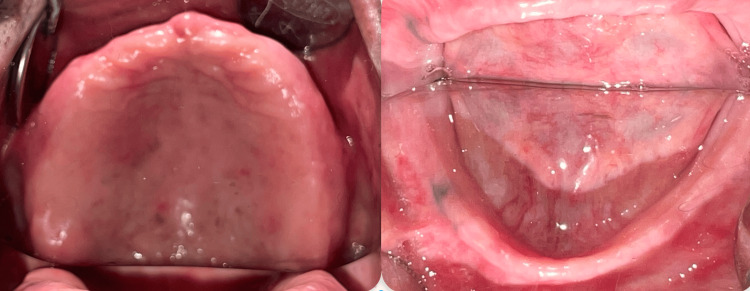
Complete edentulous maxillary and mandibular ridges of the patient.

A 64-year-old male patient reported to the Department of Prosthodontics and Crown and Bridge of Sharad Pawar Dental College and Hospital, Sawangi (M), with the chief complaint of ill-fitting dentures. The patient had been a denture wearer for one month and was finding difficulty with speech and mastication while using the denture. A thorough case history was taken, and the patient gave a history of PD for four years, with decreased neuromuscular coordination, slight facial muscle rigidity, and decreased salivation. Upon intra-oral examination, a completely edentulous maxillary and mandibular ridge, with the mandibular ridge being moderately resorbed, was seen. An increase in masticatory muscle tonicity was palpated. The ridges were free of flabby tissue and bony spicules (Figure 1).

**Figure 2:**
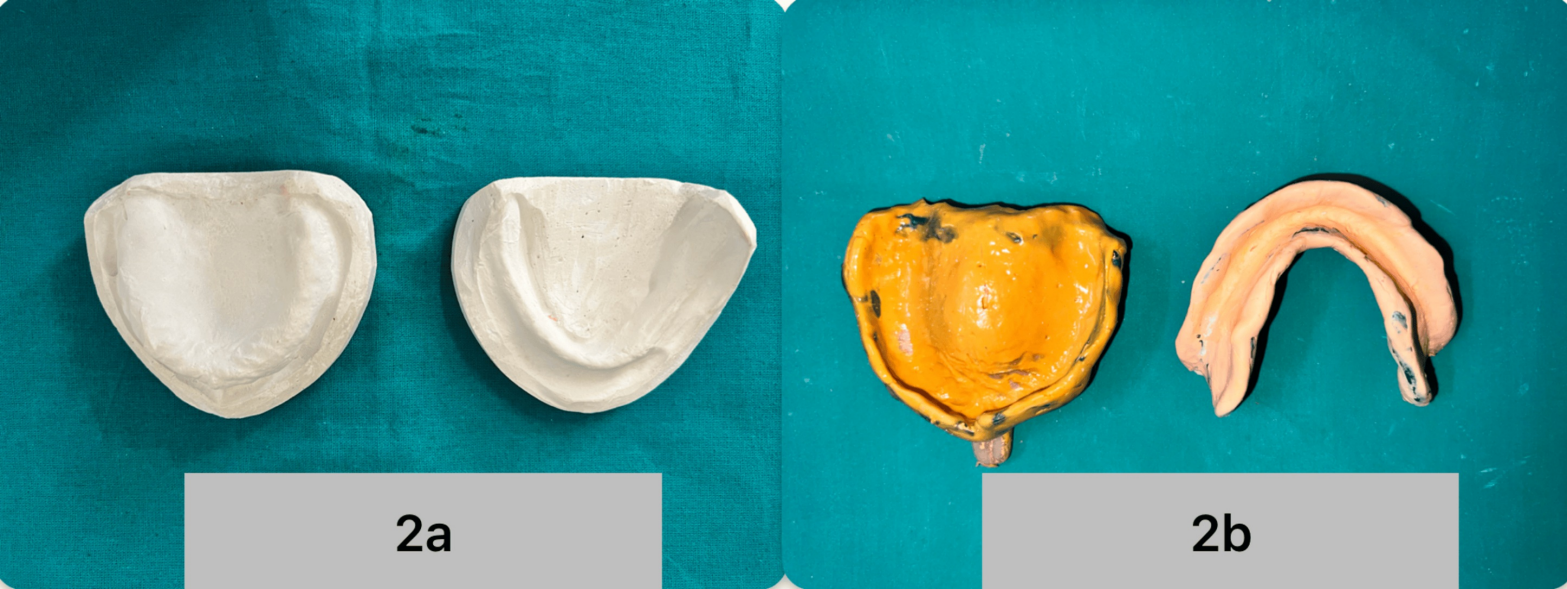
a) Primary cast of both maxilla and mandible. b) Final impression of both maxilla and mandible.

Considering the decreased neuromuscular movements and increased muscle tonicity, teeth arrangement was planned using the neutral zone concept to counter the dislodging forces experienced due to tongue and cheek movements. The preliminary impression was made using rigid, thermoplastic impression material (MDM Impression Compound) by the muco-compressive technique, followed by the primary cast fabrication in type II dental plaster. Spacer wax (Maarc Spacer Wax) was adapted over the relief areas in the maxillary and mandibular cast. The I spacer was adapted in the incisive papilla and mid-palatine raphe region, followed by a complete spacer with tissue stops over it to provide space for a wash impression on the maxillary cast. A complete spacer with tissue stops was also adapted over the mandibular cast. A custom tray was fabricated using polymethylmethacrylate (DPI RR Cold Cure) over the spacer. The final impression was made using a green stick (DPI Pinnacle Greenstick Compound) for border molding and additional silicone (Zhermack Elite HD+ Light Body Normal Set) for the wash impression. Here, due to the muscular rigidity, thorough border molding was avoided (Figures 2a-2b).

**Figure 3:**
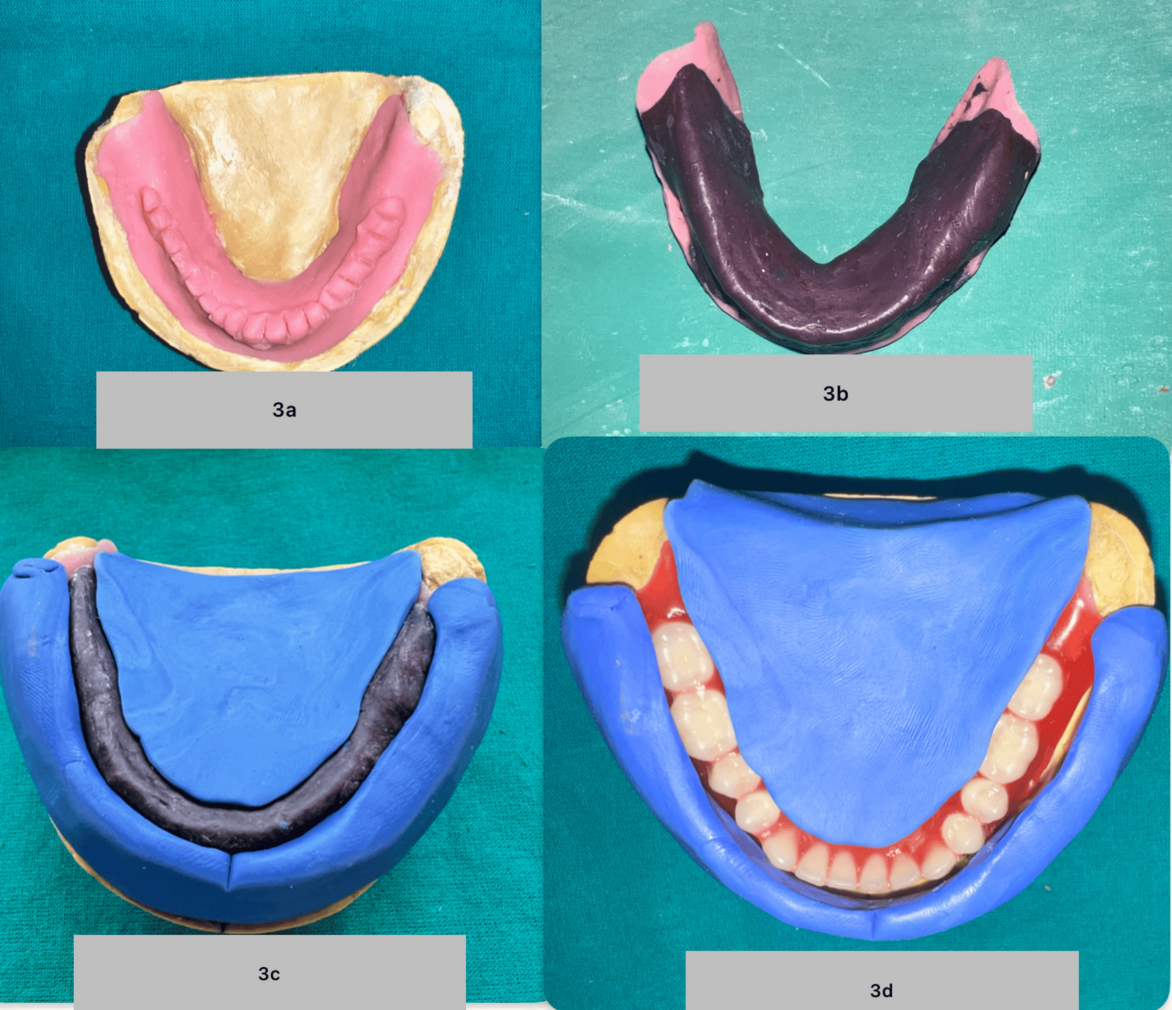
a) Temporary record base with elevated indented grooves. b) Record base loaded with the McCord and Tyson technique of admix compound. c) Neutral zone putty index. d) Teeth arrangement with the putty index guide.

On the master cast, polymethylmethacrylate (DPI RR Cold Cure) was used to fabricate a temporary record base with elevated, indented grooves for recording the neutral zone. Record base loaded with the McCord and Tyson technique of admix compound (seven-part green stick and three-part impression compound) were used. The admix was customized to record the vertical jaw relationship. Post-jaw relation recording, the jaw relation was mounted on a mean value articulator. The admix was softened and inserted in the patient’s mouth. The patient was asked to perform various cheek and tongue movements to record the neutral zone. Putty indexes (Zhermack Hydrorise Putty Fast Set) were made by adapting putty around the recorded neutral zone. The mandibular occlusal rim was modified to fit inside the putty-indexed neutral zone. Teeth arrangement was done on the mounted articulator using the putty indexes as guidance (Figures 3a-3d).

**Figure 4:**
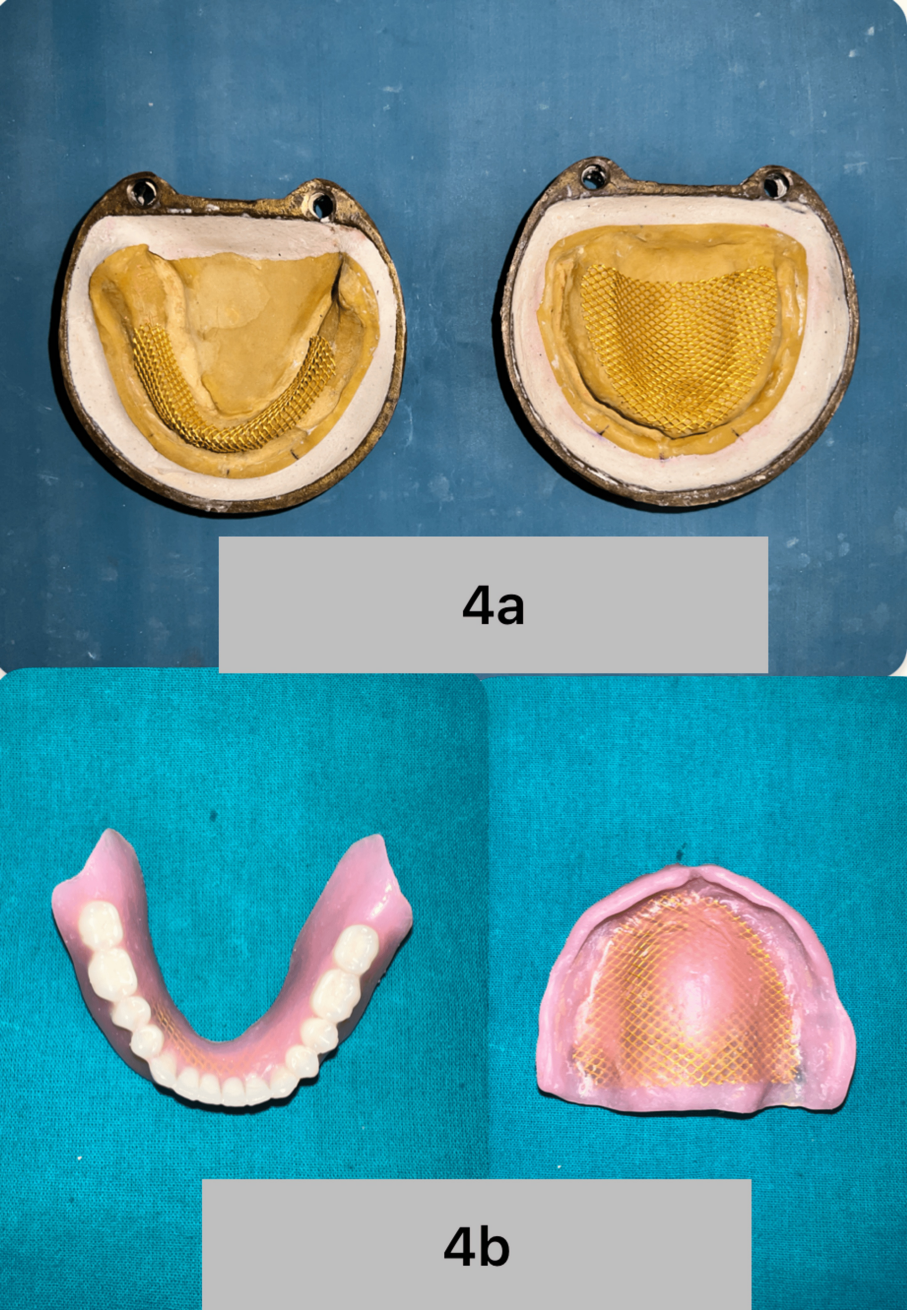
a) Metallic mesh placement before packing. b) Metallic mesh incorporated complete dentures.

Denture characterization was done by providing root anatomy wax carving. To increase the strength of the dentures to counter the impact fatigue (due to accidental fall), considering the Parkinson’s condition, a metallic mesh (Jinguang Denture Reinforcement Mesh ) was incorporated while packing the dentures with High-Impact Denture Base Resin (Dentsply Lucitone 199 Denture Base Resin). After this metallic mesh was incorporated in the complete denture packing, curing, finishing, and polishing were done in the conventional manner (Figures 4a-4b).

**Figure 5:**
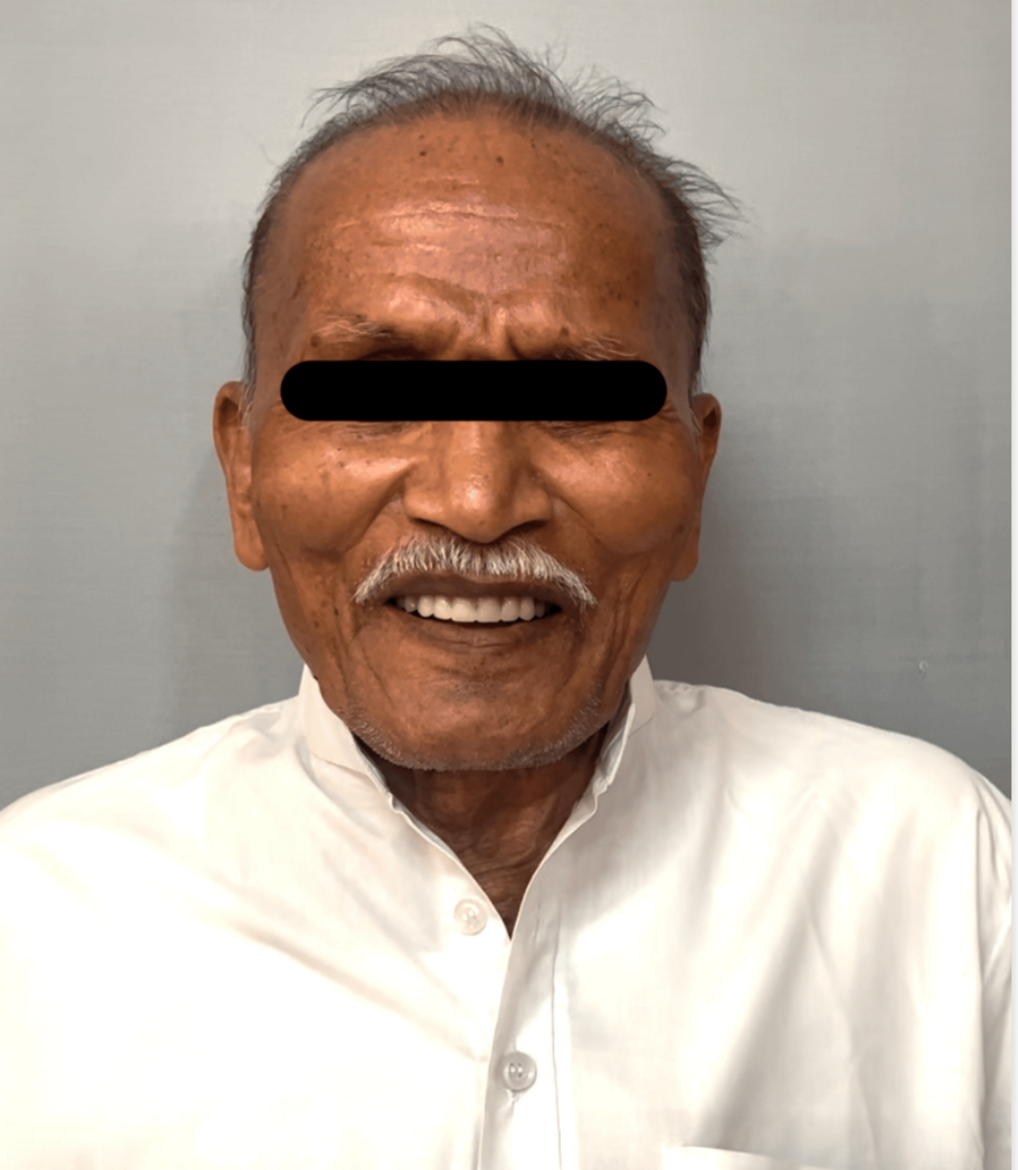
Clinical picture of the patient post-denture insertion

The clinical photograph of the patient post-denture insertion is shown in Figure 5.

## Discussion

PD, often age-related, can be debilitating for the patient. Being unable to perform daily activities can add to the psychological stress [7]. We aim for a holistic treatment plan where we not only rehabilitate the edentulous state but also achieve a comfortable fit and aesthetic appeal for the patient. In the course of border molding, the compromised neuromuscular coordination and stiffness of muscles presented a great obstacle. The best course of treatment was to go slow with the treatment, getting the patient adjusted and familiar with the various facial movements. The arrangement of teeth following the neutral zone concept reduces the dislodging forces exerted by the tongue and cheeks, thereby increasing the retention and stability of the dentures [8].

The polymethylmethacrylate commonly used for the fabrication of record bases is itself modified with an elevated intended surface to load the admix, making it cost- and technique-effective. Further, as reported in the literature, most patients with PD have rhythmic muscle contractions. Sometimes, the severity of the contractions makes it difficult to insert and remove the removable prosthesis due to decreased manual dexterity [9]. Cracks in the midline of dentures were observed in 61% of cases using mandibular dentures and in 46.87% of cases involving maxillary full dentures. Denture fracture can be caused by a variety of reasons, such as flexural fatigue from cyclic deformation and variables that change the base's stress distribution [10]. Incorporating metallic mesh into the complete denture prosthesis and using High-Impact Denture Base Resin increases its fracture resistance in the event of an accidental fall.

## Conclusions

Rehabilitating patients with a completely edentulous state of mouth can be challenging when we need to take into consideration systemic disease conditions like PD. A thorough history-taking and clinical examination should be done to identify the problem areas of the patient. Accordingly, early/late appointments should be scheduled, and modifications to the conventional way of treating patients should be made to be tailored to the patient’s requirements. Both the physical and psychological needs of the patient should be addressed appropriately.
